# From Lab to Technical CO_2_ Hydrogenation Catalysts: Understanding PdZn Decomposition

**DOI:** 10.1021/acsami.2c19357

**Published:** 2023-01-23

**Authors:** Pierfrancesco Ticali, Davide Salusso, Alessia Airi, Sara Morandi, Elisa Borfecchia, Adrian Ramirez, Tomás Cordero-Lanzac, Jorge Gascon, Unni Olsbye, Finn Joensen, Silvia Bordiga

**Affiliations:** † Department of Chemistry, NIS Center and INSTM Reference Center, University of Turin, Turin10125, Italy; ‡ King Abdullah University of Science and Technology, Thuwal23955, Saudi Arabia; § SMN Centre for Materials Science and Nanotechnology, Department of Chemistry, University. of Oslo, Sem Sælands vei 26, 0371Oslo, Norway; ∥ Haldor Topsøe, A/S Kgs., Lyngby2800, Denmark

**Keywords:** CO_2_ conversion, PdZn alloy, hydrogenation, heterogenous catalysis, zeolites, scale up

## Abstract

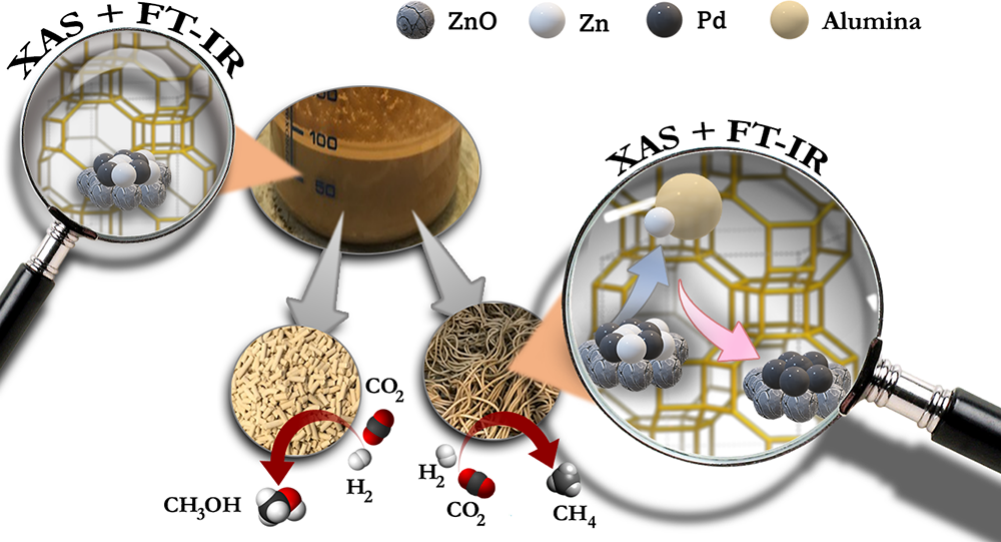

The valorization of CO_2_ to produce high-value chemicals, such as methanol and hydrocarbons, represents key technology in the future net-zero society. Herein, we report further investigation of a PdZn/ZrO_2_ + SAPO-34 catalyst for conversion of CO_2_ and H_2_ into propane, already presented in a previous work. The focus of this contribution is on the scale up of this catalyst. In particular, we explored the effect of mixing (1:1 mass ratio) and shaping the two catalyst functions into tablets and extrudates using an alumina binder. Their catalytic performance was correlated with structural and spectroscopic characteristics using methods such as FT-IR and X-ray absorption spectroscopy. The two scaled-up bifunctional catalysts demonstrated worse performance than a 1:1 mass physical mixture of the two individual components. Indeed, we demonstrated that the preparation negatively affects the element distribution. The physical mixture is featured by the presence of a PdZn alloy, as demonstrated by our previous work on this sample and high hydrocarbon selectivity among products. For both tablets and extrudates, the characterization showed Zn migration to produce Zn aluminates from the alumina binder phase upon reduction. Moreover, the extrudates showed a remarkable higher amount of Zn aluminates before the activation rather than the tablets. Comparing tablets and extrudates with the physical mixture, no PdZn alloy was observed after activation and only the extrudates showed the presence of metallic Pd. Due to the Zn migration, SAPO-34 poisoning and subsequent deactivation of the catalyst could not be excluded. These findings corroborated the catalytic results: Zn aluminate formation and Pd^0^ separation could be responsible for the decrease of the catalytic activity of the extrudates, featured by high methane selectivity and unconverted methanol, while tablets displayed reduced methanol conversion to hydrocarbons mainly attributed to the partial deactivation of the SAPO-34.

## 1 Introduction

The Sixth Assessment Report released by IPCC^1,2^ demonstrates that human activity is responsible for the increasing concentration of greenhouse gases in the atmosphere. As a matter of fact, the electricity production is still dependent on oil, natural gas, and coal, which are the main energy sources and continue to endanger the environment. Even though these energy sources strongly contribute to CO_2_ emissions in the atmosphere, they still represent an important source in several parts of the world. According to the IEA,^3,4^ contribution from fossil fuels to the world energy supply is still increasing. In 2018, they accounted for nearly 40% of global emissions and coal was responsible for about 29% of energy-related emissions. Hence, it is clear how significant reducing their consumption is, as well as mitigating the environmental consequences of their utilization. Among all the activities humanity could implement, carbon capture and utilization (CCU) is one of the best options.

To date, several research groups, start-ups and companies head toward processes aiming at achieving efficient CO_2_ reactivity with hydrogen to produce organic molecules such as methanol (MeOH) and possibly transform them into hydrocarbons by the means of a bifunctional catalyst. In particular, propane is a key feedstock molecule and strictly linked to the propylene market.

To produce propane from carbon dioxide, several works^5−8^ described metal oxide systems as supporting materials for other phases, physical mixtures, and solid solutions that could be involved in CO_2_ hydrogenation. Among the most studied metal oxides,^9,10^ some research groups focused on Zn in combination with ZrO_2_^11,12^ or Pd in combination with Zn, supported on oxidic support like CeO_2_ or similar.^7,8^ Zn is well-known to play a key role in heterolytic H_2_ splitting due to its capability in producing oxygen vacancies,^12−16^ Moreover, the presence of Pd seems to improve the activity of these catalysts in adsorbing CO_2_ and enhancing CO_2_ to methanol conversion, by alloying with Zn.^7,8^

Furthermore, ZrO_2_ is reported as an excellent support for this kind of catalysts.^11^ ZrO_2_ has been studied in many systems as a supporting material.^17^ In fact, ZrO_2_ has weak hydrophilic properties, enhances reaction products and selectivity, can bind reaction intermediates, stabilizes active species,^12,18^ reduces water adsorption,^9,19^ and enhances H_2_ dissociation.^20,21^

To convert methanol into hydrocarbons, some authors proposed bifunctional catalysts made up of an oxidic phase and a zeolite.^22,23^ According to the acidity and the type of the zeolite involved in the reaction, methanol can be converted to different hydrocarbons with a different number of carbon atoms.^24,25^

Taking all these suggestions into account, we previously proposed a PdZn@ZrO_2_ + SAPO-34 bifunctional catalyst,^26^ which demonstrated to be highly selective toward propane (>50% selectivity) with a conversion close to 40% at 350 °C, 50 bar, and 1500 mL g^–1^ h^–1^. According to our previous results, the intimate contact between the metal oxide phase and the zeolite triggers methanol to quickly react and push the equilibrium toward propane production. This type of catalyst featured the formation of a PdZn alloy surrounded by a polycrystalline and defective ZnO shell during the activation in hydrogen, which was linked to the methanol production. Instantly, the MeOH formed on the PdZn@ZrO_2_ reacted over the SAPO-34 to produce propene following the methanol-to-olefin (MTO) process while avoiding the fast deactivation due to the high partial pressure of H_2_ in the reaction medium.^27^ The presence of Pd and this hydrogen induced the hydrogenation of propene to propane, which resulted to be the main product.

Despite the outstanding performance of physical mixtures at the laboratory scale, process scale-up will require the development of technical catalysts, able to work in multiple reaction cycles and with sufficient thermal and mechanical resistances. For this purpose, active phases are commonly mixed with binders and inert fillers in a certain optimal composition, which may unfortunately cause modifications on catalytic performance.^28^ In this work, we explored the catalytic performance of different technical catalysts, prepared by scaled up synthesis routes. Characterization of the stand-alone PdZn@ZrO_2_ and combined PdZn@ZrO_2_ + SAPO-34 catalyst in two different shapes were used to understand the different behaviors of each prepared technical catalyst. Spectroscopic characterization of these systems was performed by infrared spectroscopy (FT-IR) and X-ray absorption spectroscopy (XAS). In the case of tablets, PdZn alloy formation was observed, as already found for the lab-scale catalyst in our former works.^26^ However, less selectivity to C_3_ products was achieved, likely due to the loss of SAPO-34 activity, with observable amounts of unreacted methanol. On the other hand, extrudates did not show any PdZn alloy and half of the propane selectivity was observed when compared with the lab-scale catalyst. Further investigation demonstrated the presence of zinc aluminates due to the use of Al_2_O_3_ in the preparation of the extrudates, hindering the catalytic activity.

## 2 Materials and Methods

### 2.1 Catalyst Preparation

Catalysts were prepared and shaped to form both tablets and extrudates. The PdZn@ZrO_2_ precursor, PZZ-ox, was prepared by aqueous incipient wetness impregnation of Zn- and Pd-nitrate on ZrO_2_ (Aldrich), followed by drying and calcination at 500 °C for 5 h. The calcined catalyst precursor was mixed with SAPO-34 (China Catalyst Holding Co.) and alumina binder and pelletized/tableted to form a robust catalyst body (PZZ-ox-tab). The SAPO-34 was previously calcined at 475 °C for 2 h to remove the template and carbonaceous residues in the as-received material. Final composition of the tablets was approx. 45:45:10 wt % of PdZn@ZrO_2_, SAPO-34, and Al_2_O_3_, respectively. Extrudates were prepared by mixing the calcined catalyst precursor with the SAPO-34 and alumina binder with water to form a paste. The paste was extruded on a piston extruder, dried, and calcined 5 h at 500 °C to obtain a catalyst, PZZ-ox-ext, with approx. 30:30:40 wt % of PdZn@ZrO_2_, SAPO-34, and Al_2_O_3_, respectively. For both tablets and extrudates, a dual strategy was pursued, using calcined (PZZ-ox) and reduced (PZZ-red) precursors, respectively. Reduction of the (calcined) PdZn@ZrO_2_ precursor was performed in a flow of hydrogen (2 vol %) in Ar at 400 °C for 20 h. The final catalysts with reduced precursors were subjected to final calcination as described above to produce PZZ-red-ext (extrudates) and PZZ-red-tab (tablets), except for a small sample, PZZ-red-tab-unc, which was tableted using the reduced precursor, PZZ-red, but not calcined, to be used in spectroscopic studies.

### 2.2 Activity Measurement

Catalytic tests were carried out in a 4 channel Flowrence from Avantium. The gas feed composition was 24 vol % of CO_2_, 72 vol % of H_2_, and 4 vol % of He as internal standard. We aimed to 6000 mL/g/h per channel. Prior to feeding the reaction mixture, all samples were reduced *in situ* with a pure H_2_ atmosphere for 4 h at 400 °C. The reaction temperature was set at 350 °C and the pressure at 30 bar. The reaction products were analyzed online in a gas chromatograph Agilent 7890B with two sample loops. One sample loop goes to the TCD channel with a 2 HayeSep precolumn and MS5A, where He, H_2_, CH_4_, and CO are separated. Another sample loop goes to an FID with an Innowax precolumn, a GasPro column, and another Innowax column. The GasPro column separates C1–C8, paraffins, and olefins, while the Innowax column separates oxygenated and aromatics.

Conversion (*X*) and selectivity (*S*) are reported on C1 basis and are defined as follows:
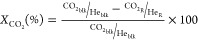

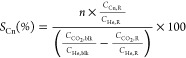
where *C*_i,blk_ and *C*_i,R_, are the concentrations determined by GC analysis in the blank and in the reactor outlet, respectively, and *n* is the number of carbon atoms of each C_n_ product. Carbon balance closure was better than 2.5% in all cases.

### 2.3 Structural and Spectroscopic Characterization

The acidity of the samples was measured via NH_3_-TPD, using a TGA/DSC1 instrument (Mettler Toledo). In a typical analysis, 70 mg of the sample was degassed at 500 °C under a 31%He–69%Ar flow rate of 75 mL/min for 110 min at a heating rate of 20 °C/min. Next, the sample was cooled to 150 °C and then saturated with a mixture of 29%He–69%Ar and 2% ammonia (2% NH_3_/29%He/69%Ar) for 30 min. The sample was then purged with a 31%He–69%Ar flow for 233 min, to remove weakly and physically adsorbed NH_3_ on the surface of the catalyst. After this operation, the sample was cooled to 140 °C, kept at that temperature for 10 min, and heated back to 150 °C and then to 600 °C at a rate of 10 °C/min, under a flow of 31%He–69%Ar carrier gas (75 mL/min). The amount of ammonia was measured as mass loss recorded as a function of temperature and time.

The textural properties of the materials were determined from the Ar adsorption isotherm in liquid argon (−186 °C) measured on a Quantachrome Autosorb iQ apparatus with the lowest pressure at *P*/*P*_0_ = 10–6. The sample (100 mg) was degassed in vacuum for 16 h at 27 °C prior the analysis. The data were fitted using an NLDFT model based on a cylindrical pore model using ASiQwin software.

This model is fit for zeolites with cylindrical pore channels such as mordenite, ZSM-5, etc. The applicable pore range is 0.35 to 100 nm.

Catalyst samples were investigated by PXRD (PanAlytical X’Pert Pro) in Bragg–Brentano geometry in reflectance mode using a Cu Kα radiation source (λ = 1.541 Å) at ambient conditions. The scan range was 5–70° with a step size of 0.017°. Rietveld refinement was performed using Topas software provided by Bruker.

Wavelength-dispersive X-ray spectrometry was performed on an FEG-EPMA JEOL JXA-8530F electron microprobe (*d* = 1 μm) operated at 20 kV, from 20 to 55 nA on cross sections of shaped samples, embedded in epoxy and ground with silicon carbide.

Zn K-edge XAS spectra were collected on self-supporting wafers (diameter 1.3 cm^2^) obtained by grinding and pressing the different catalysts in transmission mode using a Si(111) double-crystal monochromator at the BM31 beamline of the European Synchrotron Radiation Facility (ESRF). Spectra were measured in the 9.5–10.2 keV energy range with a 0.5 eV energy step and an integration time of 0.1 s/point resulting in ca. 2.5 min/scan. Six consecutive scans/sample were averaged after energy alignment to Zn metal foil, background subtraction, and jump normalization conducted with the Athena software from the Demeter Package.^29^ Spectra of reference ZnO (Honeywell, >99%) was also measured in the form of a self-supporting pellet, while the spectra of the Zn(10%)–Al_2_O_3_ model system were previously measured as described elsewhere.^30^ These two spectra were used as references (μ^ref^(*E*)) for a linear combination fitting (LCF) analysis of the sample spectra. LCF was performed with the ATHENA software trying to minimize the *R*-factor defined as
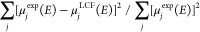
where *j* represents each experimental point in the fit-range (9645–9695 eV). μ^LCF^(*E*) was obtained by fitting μ^exp^(*E*) as linear combination of two reference spectra μ_*i*_^ref^(*E*) : μ^LCF^(*E*) = *w*_1_ μ_1_^ref^(*E*) + *w*_2_ μ_2_^ref^(*E*). LCF was performed imposing 0 ≤ *w_i_* ≤ 1 but without constraining . *R*-factor tendency to 0, i.e., μ^exp^(*E*) = μ^LCF^(*E*) and the best fit values of  were used as fit-quality indicators.

IR spectra were collected on preactivated self-supporting s wafers inserted in a home-made quartz cell with KBr windows suitable for *ex situ* and *in situ* experiments. The pellet activation procedure consisted of the following steps: (i) heating (5 °C/min) under vacuum (5 × 10^–4^ mbar) up to 400 °C, (ii) 1 h reduction at 400 °C under H_2_ (50 mbar), and (iii) outgassing and cooling down to room temperature. The IR spectra were acquired in transmission mode with a Bruker Vertex 70 spectrophotometer equipped with an MCT cryodetector working at 20 KHz, collecting 32 scans for each spectrum with 2 cm^–1^ of resolution. Under acquisition, the samples were kept under vacuum (5 × 10^–4^ mbar) through connection with a vacuum glass-line. The same line was used to dose carbon monoxide (CO) for the *in situ* experiment at RT. Spectra were acquired at increasing equilibrium pressure (e.p.) of CO up to 30 mbar.

## 3 Results and Discussion

### 3.1 Catalytic Performance of the Technical Catalysts

**Figure 1:**
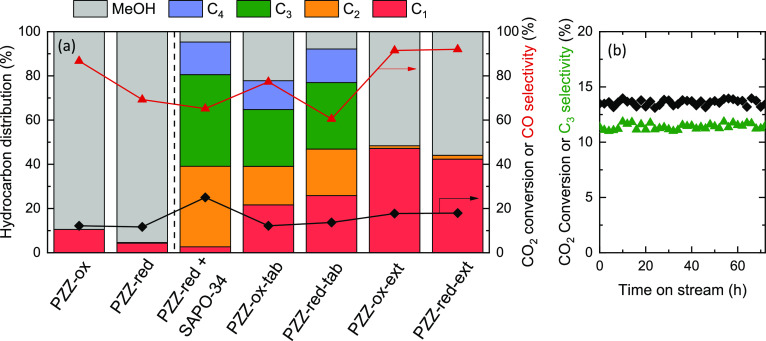
(a) CO_2_ conversion and hydrocarbon distribution for the different technical catalysts and (b) evolution with time on stream of CO_2_ conversion and C_3_ selectivity for the PZZ-red-tab catalyst. Reaction conditions: 350 °C, 30 bar, 6000 cm^3^/g/h, 1:3 CO_2_:H_2_.

A summary of the scaled-up catalysts, synthesized in tablets and extrudates, is shown in Table 1, where a brief description of the preparation protocol and basic properties are detailed. For the sake of comparison, the commercial SAPO-34 used as the acid function in the technical catalyst is also included in the table. All these samples were tested in the tandem hydrogenation of CO_2_ to hydrocarbons under typical conditions for this catalyst according to our previous results.^26^Figure 1a shows the CO_2_ conversion and the product distribution obtained with the technical catalysts. Please note that PZZ-ox and PZZ-red consist of a scaled up and optimized synthesis protocol, which was previously reported elsewhere.^26^ These two scaled up stand-alone PdZn@ZrO_2_ do produce methanol and CO, in line with the laboratory-scale PdZn@ZrO_2_ catalyst. Nevertheless, the PZZ-red catalyst, which was reduced *ex situ* after the synthesis, performed ostensibly better, with similar conversion and more than double methanol selectivity due to the decrease in CO production (see left panel in Figure 1a and detailed product distribution in Table S1).

**Table 1:** Summary of the Prepared Samples with Their Main Physicochemical Properties

sample name	sample description	composition (wt %)(rest to 100% is oxygen)	NH_3_ capacity (mmol/g)	surface area (Hg)(m^2^/g)	pore volume < 6 Å Ar BET (cm^3^/kg)
SAPO-34			0.70		224
PZZ-ox	precursor calcination	Pd(1.8)		16	
		Zn(8.7)			
		Zr(62.8)			
PZZ-red	PZZ-ox reduction	Pd(1.8)		15	
		Zn(8.8)			
		Zr(62.4)			
PZZ-ox-tab	PZZ-ox mixed with SAPO-34 + Al_2_O_3_, tableted, and calcined	Pd(0.7)	0.36	34	104
		Zn(3.9)			
		Zr(27.4)			
		Al(13.1)			
		Si(0.9)			
		P(9.1)			
PZZ-red-tab	PZZ-red mixed with SAPO-34 + Al_2_O_3_, tableted, and calcined	Pd(0.8)	0.35	31	106
		Zn(3.9)			
		Zr(27.4)			
		Al(12.6)			
		Si(0.9)			
		P(9.2)			
PZZ-ox-ext	PZZ-ox mixed with SAPO-34 + Al_2_O_3_, extruded, and calcined	Pd(0.5)	0.12	123	58
		Zn(2.7)			
		Zr(19.2)			
		Al(26.0)			
		Si(0.6)			
		P(5.4)			
PZZ-red-ext	PZZ-red mixed with SAPO-34 + Al_2_O_3_, extruded, and calcined	Pd(0.5)	0.12	125	57
		Zn(2.7)			
		Zr(19.5)			
		Al(26.5)			
		Si(0.6)			
		P(5.5)			

To compare the catalytic performance of the technical catalysts, we have included the catalytic results obtained with a physical mixture of the PZZ-ox catalyst and the commercial SAPO-34 in Figure 1a. Please note that the composition of each technical catalyst is slightly different, so a small decrease in CO_2_ conversion could be expected from the physical mixture to tablets and extrudates (see Materials and Methods). As observed before, this physical mixture boosts CO_2_ conversion (from ca. 12 to 25%) by converting *in situ* the produced methanol to hydrocarbons. CO selectivity is significantly reduced (from ca. 86 to 65%) due to a shift in the reaction network equilibria, and methane selectivity is also minimized. Nonetheless, this behavior was not observed for the technical catalysts. Looking at the tablet technical catalysts, both PZZ-ox-tab and PZZ-red-tab catalysts are able to yield propane as the main hydrocarbon product. In addition, PZZ-red-tab outperformed its nonreduced counterpart PZZ-ox-tab with double propane selectivity (see Table S1), as one should expect after the comparison of the mixed oxides PZZ-ox and PZZ-red. The catalytic performance of PZZ-red-tab is also very stable during the 72 h study (see Figure 1b). Nevertheless, the catalytic performance of this technical catalyst is significantly worse than the physical mixture, with a CO_2_ conversion of only ca. 14% and a high methane selectivity (10%, Table S1). This loss of activity becomes even worse when the technical catalyst is shaped into an extrudate form (PZZ-ox-ext and PZZ-red-ext). The catalytic activity is lost, without observing any propane in the product effluent. Surprisingly, the catalyst converts CO_2_ into methanol (with some DME) and methane as the main byproduct. This result suggests a very likely poisoning of the SAPO-34 sites during catalyst shaping and potential modifications of the hydrogenation function, which is still able to hydrogenate CO_2_.

### 3.2 Physicochemical Characterization of Technical Catalysts

To understand the reason and changes of the active phases during catalyst shaping, catalysts were characterized using several techniques.

Table 1 summarizes the main physicochemical properties of the studied catalysts, including their composition, surface area, and ammonia capacities. Accounting the different compositions targeted, the stand-alone PdZn@ZrO_2_ (PZZ-ox and PZZ-red) mixed oxides have Pd and Zn amounts close to the nominal values of 2 and 10 wt %, respectively. Likewise, tablet and extrudate catalysts show the corresponding values according to the concentration of the active phase in the final catalyst (45 and 30 wt % of each function in the tablets and extrudates, respectively). Regarding catalyst porous texture, the tablet catalysts (PZZ-ox-tab and PZZ-red-tab) preserve the micropore volume (<7 Å): the micropore volume of the SAPO-34 raw material is about 220 cm^3^/kg and with about 45 wt % of SAPO-34 in the tableted catalyst, a micropore volume just above 100 cm^3^/kg is reasonable. Same conclusions can be extracted from ammonia capacity: 0.35 mmol/g with 0.70 mmol/g for the SAPO-34 raw material. However, lower micropore volumes (58 and 57 cm^3^/kg) and ammonia capacities (both 0.12 mmol/g) than expected from their content of 30 wt % SAPO-34 are observed for the extrudates. By simple interpolation, expected micropore volume and ammonia capacity should be 66 cm^3^/kg and 0.20 mmol/g, respectively. Thus, a minor loss of micropore volume, possibly within the uncertainty of measurement, and a significant reduction in ammonia capacity have occurred during mixing and extrusion.

**Figure 2:**
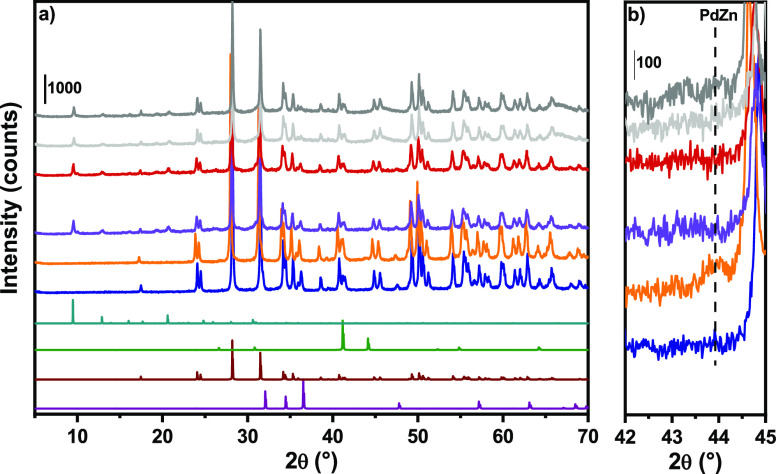
(a) PXRD patterns (bottom to top) of simulated h-ZnO (purple), m-ZrO_2_ (wine), t-PdZn (green), SAPO-34 (light blue) and experimental PZZ-ox (dark blue), PZZ-red (orange), PZZ-ox-tab (violet), PZZ-red-tab (dark red), PZZ-ox-ext (light gray), and PZZ- red-ext (dark gray). (b) Magnification of the range 42–45° to highlight the PdZn alloy reflection.

PXRD patterns in Figure 2 showed as the stand-alone PdZn@ZrO_2_ (PZZ-ox) catalysts presented PdO, ZnO, and monoclinic ZrO_2_ reflections. After catalyst reduction, ZnO and PdO reflection intensity decreased, suggesting a consumption of their phase to form a β_1_-PdZn alloy, in which the (101) reflection is visible in the PZZ-red pattern (Figure 2b).^31^ After tablet and extrudate preparation, SAPO-34 reflections were observed, while the PdZn signal disappears, suggesting that decomposition of this phase might occur. Moreover, there is a loss of ZnO content in tablets and extrudates, which is more pronounced in the second case compared to the first one. These results point to some degree of ion exchange of the SAPO-34 acid sites already taking place during the mixing and extrusion operations.

### 3.3 Morphology and Element Distribution of the Technical Catalysts

**Figure 3:**
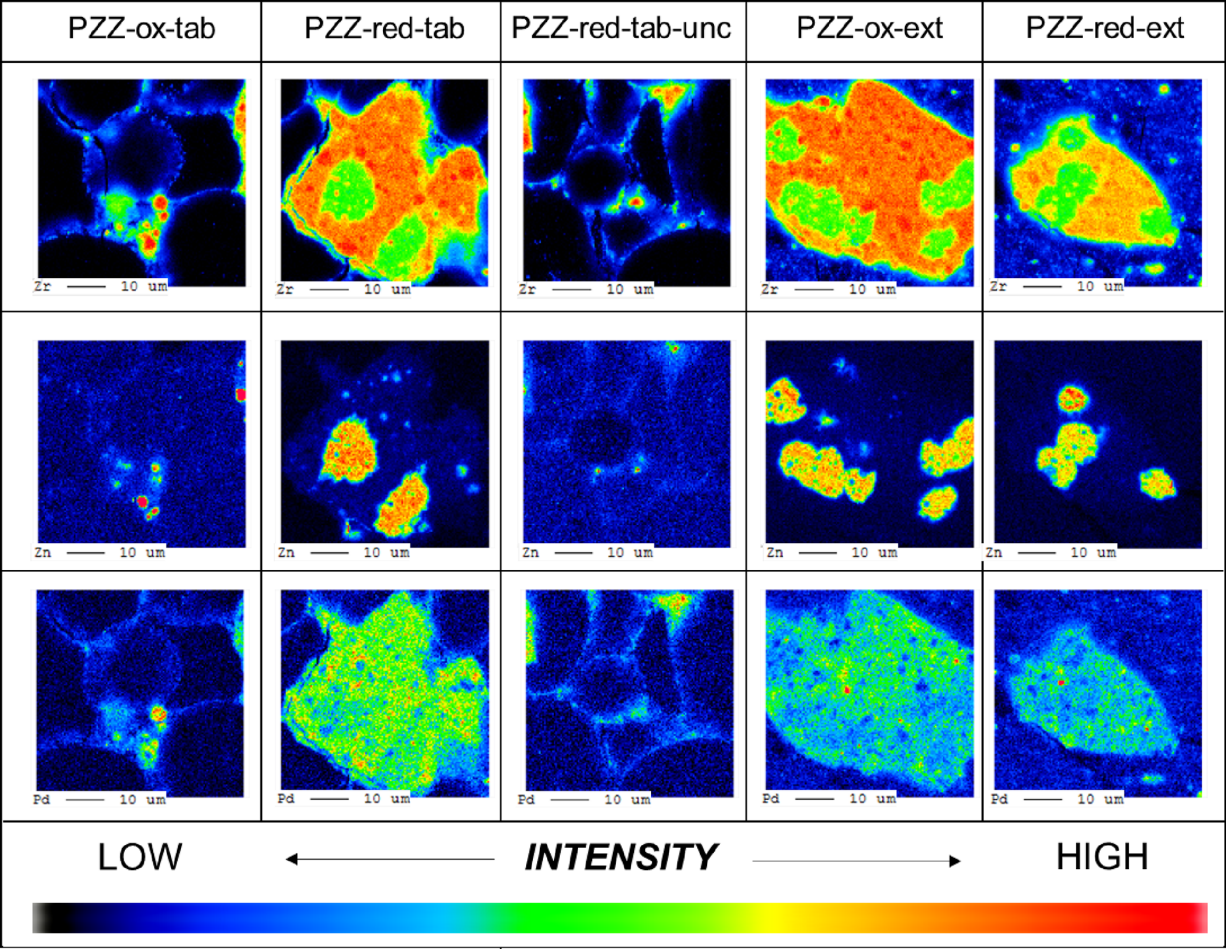
WDS intensity mapping of Zr, Zn, and Pd in dual function catalysts shaped as tablets and extrudates, applying calcined and calcined & reduced PdZn@ZrO_2_ precursors, respectively (all catalysts subjected to calcination at 500 °C subsequent to shaping, except PZZ-red-tab-unc).

**Figure 4:**
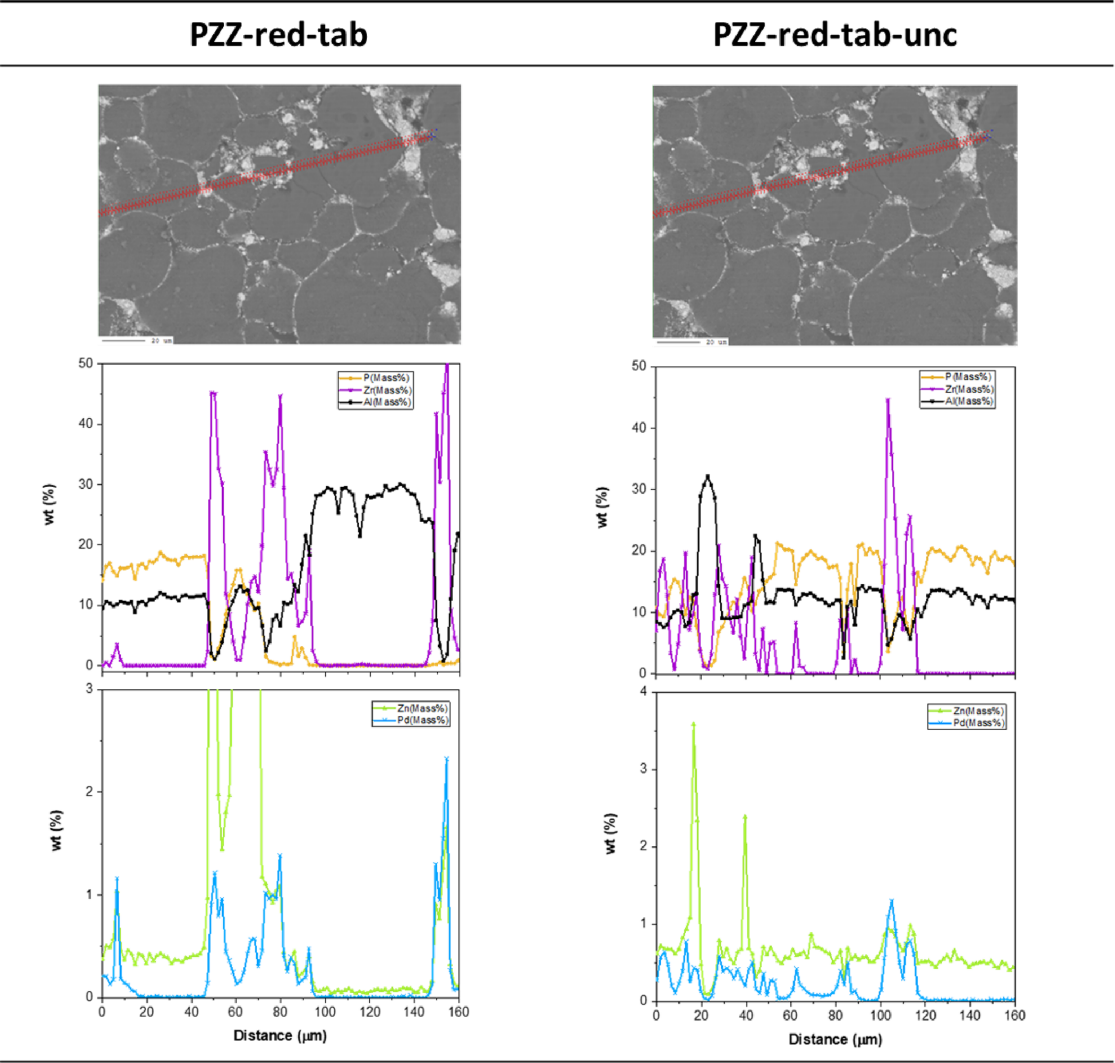
Tablet element concentration profile. PdZn@ZrO_2_ precursor: calcined after tableting (PZZ-red-tab, left) and uncalcined (PZZ-red-tab-unc, right).

**Figure 5:**
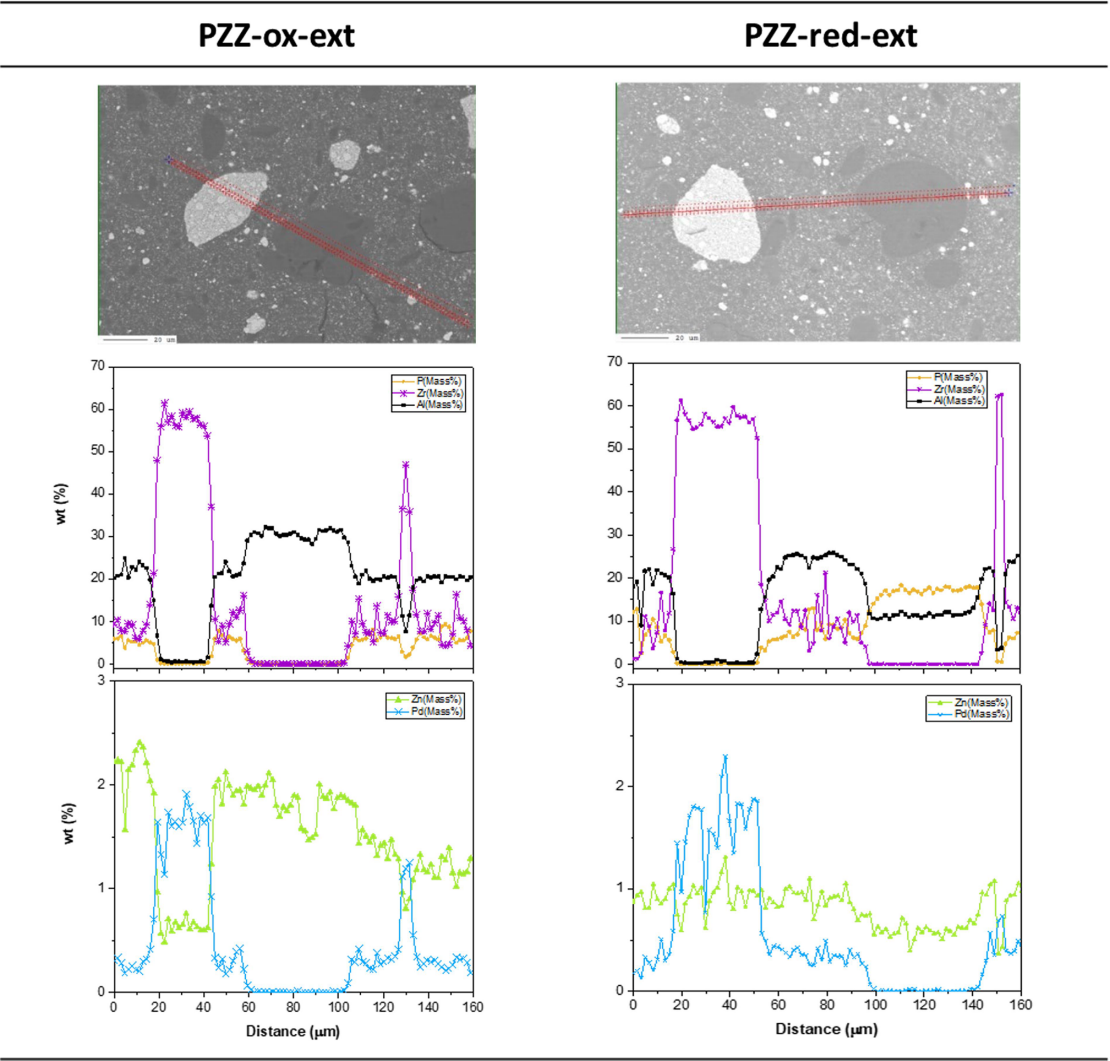
Extrudate element concentration profile. PdZn@ZrO_2_ precursor: calcined (PZZ-ox-ext, left) and calcined & reduced (PZZ-red-ext, right) prior to mixing, extrusion, and final calcination.

Selected WDS intensity mapping images of Zn, Zr, and Pd on the shaped catalysts are shown in Figure 3. The images reveal that tableted and extruded catalysts exhibit the same pattern: Zn is distributed unevenly, forming ZnO islands observed by PXRD (Figure 2), while Pd is distributed more uniformly onto the ZrO_2_ surface. The formation of ZnO islands renders a significant part of the ZrO_2_ surface covered with Pd only. We further recorded element concentration profiles along lines across the shaped catalysts, as shown in Figure 4 (tablets) and Figure 5 (extrudates). These profiles strongly support that migration of Zn is an issue irrespective of the PdZn@ZrO_2_ part of the catalyst being calcined or calcined & subsequently reduced in hydrogen prior to shaping, and whether in the form of extrudates or tablets. The profiles indicate that Zn tends to migrate onto both the alumina binder (mainly in extrudates) and the SAPO-34 phases, whereas Pd appears to be strongly adsorbed on the ZrO_2_ support, in line with the ZnO loss observed by PXRD. For the sake of completeness, it should be mentioned that no differences between PZZ-ox-tab and PZZ-red-tab could be observed (SEM micrographs shown Figure S1, including the uncalcined PZZ-red-tab version).

The uneven distribution of Zn leaves Pd partly and directly supported on ZrO_2_, as further confirmed by FT-IR spectroscopy results (*vide infra*). Pd is known as an excellent catalyst for the methanation reaction,^32,33^ both from CO and CO_2_. Therefore, the deposition of only Pd on the ZrO_2_ surface (in the absence of Zn/ZnO) may be the primary cause for the high selectivity of methane (Figure 1a) due to the impossibility to form the PdZn alloy (see results below). Also, for the sake of the process, the methanation reaction is hydrogen-intensive and should be avoided or at least minimized. In addition, the mobility of Zn/ZnO makes Zn species migrate to both the alumina and SAPO-34 phases. Apart from losing Zn from the ZrO_2_ support, migration to the SAPO-34 is likely to cause ion-exchange of acid sites, already taking place during shaping, thereby reducing acidity. It should also be noted that, apart from the significant migration of Zn in the preparative steps, Zn migration is likely to become exacerbated during operating conditions due to the presence of steam at high temperatures. SAPO-34 itself is quite steam-tolerant, but with steam facilitating and increasing the mobility of Zn, we may expect extensive ion-exchange to take place under operating conditions and regeneration, rendering the acid function catalyst gradually less and less efficient in the conversion of methanol to hydrocarbons.

### 3.4 Spectroscopic Characterization

**Figure 6:**
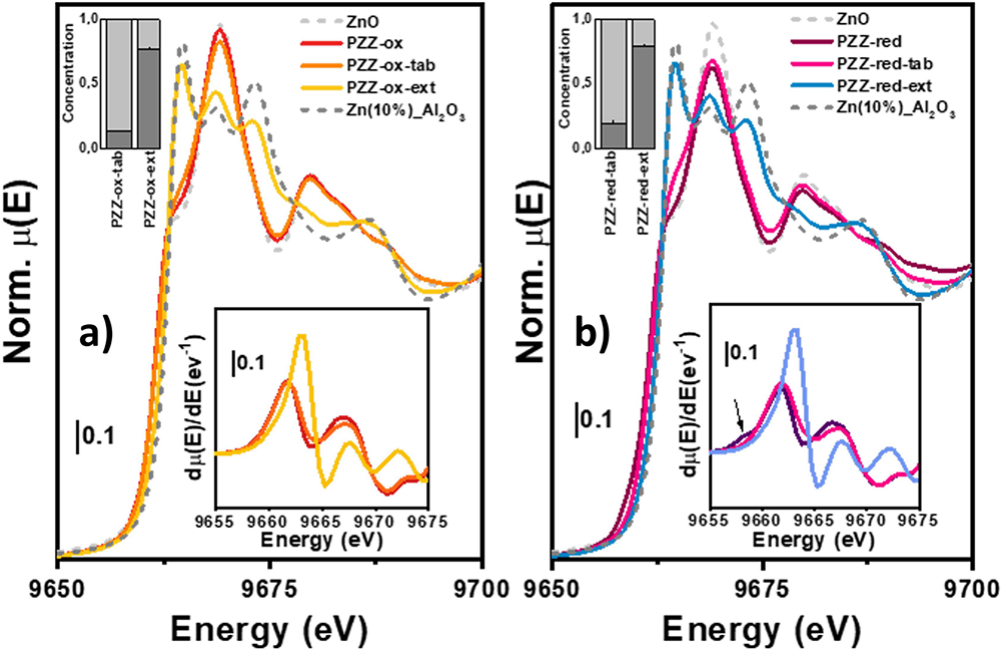
Zn K-edge EXAFS spectra for the analyzed samples, ZnO and Zn(10%)Al_2_O_3_. Panel (a) displays the oxidized samples; panel (b) displays the activated/reduced samples. Spectra first derivative and LCF results are reported in the bottom and top insets, respectively. Zn(PdZn) contribution is indicated with the arrow.

**Figure 7:**
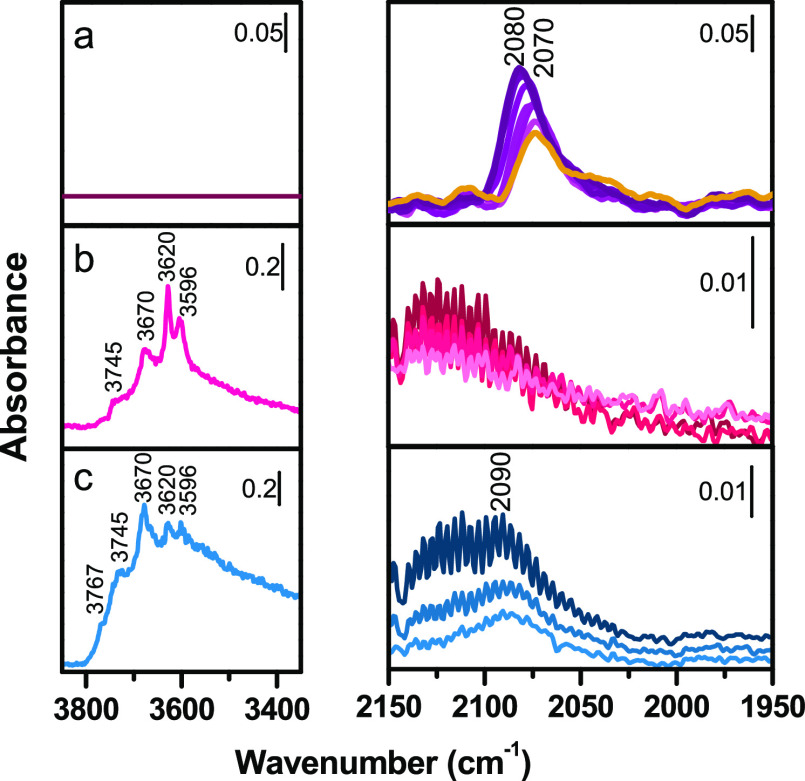
*In situ* IR spectra of (a) PdZn@ZrO_2_-red (PZZ-red), (b) PZZ-red-tab, (c) PZZ-red-ext. Left panels: OH stretching region of activated samples. Right panels: adsorbed CO stretching region of activated samples in presence of increasing CO ep (from 0.005 to 30 mbar) at RT. Darker colors indicate higher CO pressure. The yellow line in (a) refers to the PZZ–red/CO interaction after outgassing at RT.

Considering PdZn alloys, several previous studies exploited XAS to investigate Zn and Pd speciation in the employed catalysts. However, since most of relevant information concerning the Pd chemical environment was extracted by FT-IR spectroscopy of CO adsorption, we focused the XAS analysis on the Zn K-edge, which on the contrary is a silent-element from the CO viewpoint.^26,34−37^ As indicated by PXRD data and often reported in the literature, during PdZn preparation, an excess of ZnO was observed. Indeed, the PZZ-ox catalyst, prepared by precursors’ calcination, presented Zn with ZnO local geometry (Figure 6a). Even though a major fraction of Zn was present as ZnO, the catalyst obtained after PdZn@ZrO_2_-ox H_2_ thermal treatment (namely, PZZ-red) presented a small energy shift of the Zn K absorption edge, clearly observable in the spectrum first derivative (Figure 6b), previously associated to PdZn alloy formation.^26,37^ Since the PdZn fingerprint was also observed by PXRD (Figure 2) and CO adsorption (Figure 7), we can use this shift to track the PdZn presence/absence along the catalyst treatments. To guarantee catalyst activity for the methanol-to-olefin reaction, PdZn@ZrO_2_-ox/red were further mixed, tableted/extruded, and calcined with SAPO-34 and Al_2_O_3_ binder. PZZ-ox-tab, the catalyst originated from PdZn@ZrO_2_-ox despite of little variations rationalized below, presented a very similar spectrum to its precursor. On the contrary, PZZ-red-tab catalysts showed some differences in the white-line region: (I) absence of PdZn-related energy shift and (II) rise of shoulder in the lower-energy region of the spectrum. Since the Zn(PdZn) signal was still present in tablets-red-unc (see Figure S2), Zn reoxidation can be considered as a direct consequence of the catalyst calcination. A different scenario was instead observed when tablets were replaced by extrusion. PZZ-ox-ext and -red-ext, obtained by extrusion and calcination of PdZn@ZrO_2_-ox and PdZn@ZrO_2_-red with SAPO-34 and Al_2_O_3_, respectively, presented similar Zn K-edge spectra to those of Zn-aluminates observed by Pinilla-herrero et al.^30^ Moreover, since Zn migration was not observed when mixing PdZn@ZrO_2_ with SAPO-34,^26^ PZZ-ox-ext/red-ext spectra suggested that Zn aluminates are most likely formed when contacting the catalyst with an Al_2_O_3_-based binder, which confirmed the results observed before by SEM analysis (Figure 3). These fingerprints, even though very weak, can be observed also in the PZZ-ox-tab/red-tab spectra as shoulders around 9663 eV, suggesting that a minor Zn migration might take place also in tablets.

Linear combination fit (LCF) analysis of the samples using ZnO and Zn(10%)-Al_2_O_3_ references was then applied to quantify the Zn-aluminate formation, related to Zn oxide-to-binder migration phenomena. As expected, we observed that Zn migration is limited to a ∼15% total Zn by the tablet process, while it is enhanced to ∼80% total Zn by the extrusion one. This result is in line with the observed loss of activity. Indeed, some activity for the formation of hydrocarbons via methanol was observed for the pelletized/tableted technical catalysts, whereas a negligible amount of propane was shown by extrudates (Figure 1).

To investigate the nature of the surface-active sites, FT-IR spectra of CO adsorbed at RT were collected for the most relevant catalysts, i.e., PZZ-red/PZZ-red-tab/PZZ-red-ext. The spectra of PdZn@ZrO_2_-red (PZZ-red) in the presence of increasing equilibrium pressure (e.p.) of CO adsorbed at RT are reported in the right panel of Figure 7a. The diagnostic stretching region of adsorbed CO is reported subtracting the spectrum of pristine PdZn@ZrO_2_-red from those obtained in the presence of the probe molecule. The first interaction of CO with the surface generates a signal centered at 2070 cm^–1^ (lighter violet spectrum). This frequency is in line with CO adsorption on Pd^0^ sites in a PdZn alloy studied in other previous works.^26,34,38−40^ Indeed, the observed frequency is slightly lower than the one observed on metallic Pd^41^ and it can be explained by Zn-to-Pd charge transfer that leads to a strengthening of the Pd(4d)-CO(2π) backdonation, described by a downward shift of ν(CO-Pd). Moreover, no bands related to bridged carbonyls, characteristic of Pd^0^,^41^ are present below 2000 cm^–1^. These results confirm PdZn alloy formation as revealed by PXRD and XAS measurements. The blue shift observed upon CO adsorption up to 2082 cm^–1^ (darker violet spectrum) can be explained considering the so called “chemical effect”, i.e., when the surface coverage increases, CO σ-donation and π-backdonation contributions decrease, weakening the bond of CO with the site and shifting the CO vibration frequency at lower and higher frequencies, respectively, according to the dominating contribution.^41,42^

Once the sample is subjected to CO dynamic outgassing, a persistent band of interacting CO is still present and moves back in frequency in the former position due to the abruption of the “chemical effect” (Figure 7a yellow line). The band is structured by a broad component at 2070 cm^–1^, compatible with the formation of stable Pd^0^ linear carbonyls. Figure 7b reports the IR spectra collected for the bicomponent sample PZZ-red-tab. The OH stretching region of the IR spectrum of the activated powder, reported in the left panel of Figure 7b, is characterized by several signals between 3800 and 3500 cm^–1^. This contrasts with the case of sample PdZn@ZrO_2_-red (PZZ-red), where no hydroxyls signals are visible (Figure 7a, left panel). These bands are relatable to the ν(OH) bands of the typical isolated hydroxyl groups of the SAPO-34 surface, namely, Si–(OH) at 3745 cm^–1^ and P–(OH) at 3670 cm^–1^. Moreover, two very intense vibrational bands are visible at 3620 and 3596 cm^–1^ corresponding to the ν(OH) of bridged P–(OH)–Si (Bronsted acidic sites) located in two different crystallographic positions.^43^ In the right panel of the same figure, the stretching region of CO adsorbed on the activated sample at RT is reported. Due to the weak interaction with CO, no adsorption at RT on the SAPO-34 of the catalyst is expected and the probe is considered to be selective for only interacting with metal sites (Pd). The spectra, collected at increased CO e.p. (from lighter to darker pink curves), are characterized by the typical roto-vibrational profile of the free vibrating CO centered at 2143 cm^–1^. Differently from the parent PdZn@ZrO_2_-red sample, any band associated with the formation of diagnostic Pd carbonyls could not be distinguished. The missing interaction between Pd and the CO probe can be explained with the decomposition of the PdZn alloy during the catalyst preparation to reasonably produce Pd/PdO (unknown) and ZnO. ZnO likely covers all Pd sites hampering CO adsorption.^44^ Again, this observation well aligns with the XAS results that demonstrated a relevant presence of oxidized Zn in PZZ-red-tab. In order to give an explanation on the observed catalytic activity of tablets, despite the absence of the PdZn alloy, we supposed that the higher C1 (i.e., methane) amount produced from tablets rather than PZZ-red could be related to metallic Pd. Unfortunately, we had no evidence from FT-IR measurements, but we cannot exclude (i) the presence of metallic Pd on the surface of ZrO_2_ covered by a ZnO extra-phase or below the detection limit of the technique and (ii) a possible influence of ZrO_2_ support. As already mentioned, Pd is well known acting as a methanation catalyst^32,33^ and it could be the main cause of the methane production. Moreover, for extrudates, the role played by Zn-aluminates cannot be ruled out. According to the literature,^45,46^ Zn aluminates can take part to some CO_2_ hydrogenation reaction that could lead to methane production. However, ZrO_2_ has already been demonstrated to influence the activity of some systems,^47^ and thus its role could not be completely excluded.

Figure 7c reports the IR spectra of the bifunctional catalyst PZZ-red-ext. Similarly, to PZZ-red-tab, the intense ν(OH) signals of SAPO-34 hydroxyls groups are visible in the left panel of Figure 7c. In contrast to the former sample, the signals generated by defective sites (Si–OH and P–OH) are more intense than ones associated with the Brønsted sites. An additional signal is also visible at 3767 cm^–1^ generated by Al–(OH). The presence of this species may be due to point defects in the SAPO-34 framework or, reasonably, to alumina surface Al–(OH) (the binder in the catalyst formulation). The massive presence of Al_2_O_3_ also affects the Zn K-edge XAS spectrum, as previously described. The interaction of CO with the surface of PZZ-red-ext at increasing CO e.p. induces the formation of a very broad and weak carbonyl band at 2090 cm^–1^ (right panel of Figure 7c). The upward shift of ν(C≡O) with respect to that observed for the PdZn@ZrO_2_-red sample suggests the formation of linear carbonyls on metallic Pd^0^ centers not interacting with Zn, evidencing the decomposition of the alloy. This should result in the formation of bridged carbonyls as well: reasonably, taking into account the very low intensity of the band at 2090 cm^–1^ and, therefore, the very low amount of accessible Pd^0^ sites, the amount of bridged CO could be under the detection limit. Following XAS results showing Zn migration to form aluminates, we can conclude that ZnO (that covers Pd in PZZ-red-ext) is, in this case, migrated to the binder. This result further confirms the hindered formation of the PdZn alloy, which was identified as the main reason for this catalyst’s outstanding activity to methanol. Moreover, the lack of detected ZnO on the ZrO_2_ surface in close proximity to Pd sites leads to the presence of metallic Pd sites that boosts the undesired methanation reaction, clearly identified in our catalytic test result.

## 4 Conclusions

In this work, a scaled up PdZn@ZrO_2_ + SAPO-34 catalyst was investigated. On the basis of catalytic and characterization results, it was demonstrated that the catalyst is featured by a PdZn alloy as already studied for lab-scale catalysts.

The catalytic performances are remarkably influenced by the preparation method affecting and modifying the element distribution in the different phases (i.e., Zn migration in the alumina phase). Indeed, due to Zn migration, SAPO-34 poisoning followed by the deactivation of the catalyst could explain the activity loss and cannot be ruled out. Explaining that the Zn migration is not straightforward, but on the basis of known literature data concerning Zn volatility and Zn-aluminate formation, we think that every high-temperature treatment influenced the element distribution inside the sample and the presence of Al_2_O_3_ as a binder worsened the entire scenario. The combination of all these phenomena could represent a driver for Zn migration. For instance, both the tablets and the extrudates showed the presence of Zn aluminates even before the activation, and the concentration increased upon this treatment, suggesting that the migration occurred during these steps (as reported in detail in Table S2 in the Supporting Information). Comparing the as-prepared samples of both tablets and extrudates, the higher amount of Zn aluminates produced in the extrudates and observed before the activation step suggests that for this sample, both the synthesis protocol and the calcination could have strongly contributed to the migration.

Comparing the results obtained by PZZ-red+SAPO-34 with tablets (PZZ-red-tab) and extrudates (PZZ-red-ext), a variation in the hydrocarbon distribution was observed. PZZ-red-tab did not show the presence of the alloy and its worse catalytic performances could be linked to the presence of a ZnO extra-phase on ZrO_2_ support. As reported by XAS LCF, a higher amount of Zn aluminates is observed after reduction, which could contribute to the observed C1 and MeOH production.

As for PZZ-red-ext, the PdZn alloy is not present, the lack of conversion could be mainly explained by SAPO-34 poisoning, but the Zn migration into the alumina phase could also have contributed. XAS LCF showed the highest amount of Zn aluminates and subsequent poisoning of SAPO-34. Moreover, FT-IR measurements following CO adsorption demonstrated the presence of metallic Pd. Hence, the high amount of methane in the hydrocarbon distribution could be explained by the capability of Pd to produce methane from CO_2_ or CO, in addition to the production of methanol molecules.

The results of the study may be interesting to highlight the critical point that could emerge during the development of scaled up bifunctional catalysts for CO_2_ hydrogenation reactions.

## Supporting Information Available

Additional experimental results, including catalytic performances of the different scaled-up samples, SEM images, and additional XAS data related to the linear combination fit (PDF)
