# Supplementary material for: From Lab to Technical CO2 Hydrogenation Catalysts: Understanding PdZn Decomposition
Source: ACS Appl Mater Interfaces. 2023 Jan 23;15(4):5218–28. doi: 10.1021/acsami.2c19357 (PMC9906622; doi:10.1021/acsami.2c19357)
Supplement: Supplementary file 1 — am2c19357_si_001.pdf [file am2c19357_si_001.pdf]

Supporting Information

From lab to technical CO_2 hydrogenation catalysts: understanding PdZn decomposition

Pierfrancesco Ticali[a], Davide Salusso[a], Alessia Airi[a], Sara Morandi[a], Elisa Borfecchia[a], Adrian Ramirez[b], Tomás Cordero-Lanzac[c], Jorge Gascon[b], Unni Olsbye[c], Finn Joensen[d], Silvia Bordiga[a*]*

[a]University of Turin, Department of Chemistry, NIS Center and INSTM Reference Center, Turin 10125, Italy.

[b]King Abdullah University of Science and Technology, Thuwal 23955, Saudi Arabia

[c] SMN Centre for Materials Science and Nanotechnology, University of Oslo, Sem Sælands vei 26, 0371 Oslo, Norway

[d]Haldor Topsøe, Kongens Lyngby, Denmark.

Prof. Silvia BORDIGA – e-mail: silvia.bordiga@unito.it

Dr. Finn JOENSEN – e-mail: FJ@topsoe.com

Table S1. Overview of the catalytic performance of the different scaled-up samples. Reaction conditions: 350 C, 30 bar, 6000 $cm^3/g/h$, 1:3 CO_2:H_2.

		Selectivity									
**Sample**	**Conv. (%)**	**CO**	**MeOH**	**DME**	**CH4**	**C2**	**C2=**	**C3**	**C3=**	**C4**	**C4=**
**PZZ-ox**	12.18	86.72	11.67	0.21	1.4	0.01	0.00	0.00	0.00	0.00	0.00
**PZZ-red**	11.68	69.25	28.55	0.79	1.32	0.09	0.00	0.00	0.00	0.00	0.00
**PZZ-ox-tab**	12.15	77.28	3.26	1.75	4.87	3.93	0.01	5.79	0.01	2.24	0.7
**PZZ-red-tab**	13.66	60.52	2.28	0.8	10.08	8.2	0.01	11.65	0.09	4.31	1.6
**PZZ-ox-ext**	17.71	91.47	2.87	1.53	4.02	0.10	0.00	0.00	0.00	0.00	0.00
**PZZ-red-ext**	17.91	92.02	2.91	1.56	3.38	0.14	0.00	0.00	0.00	0.00	0.00

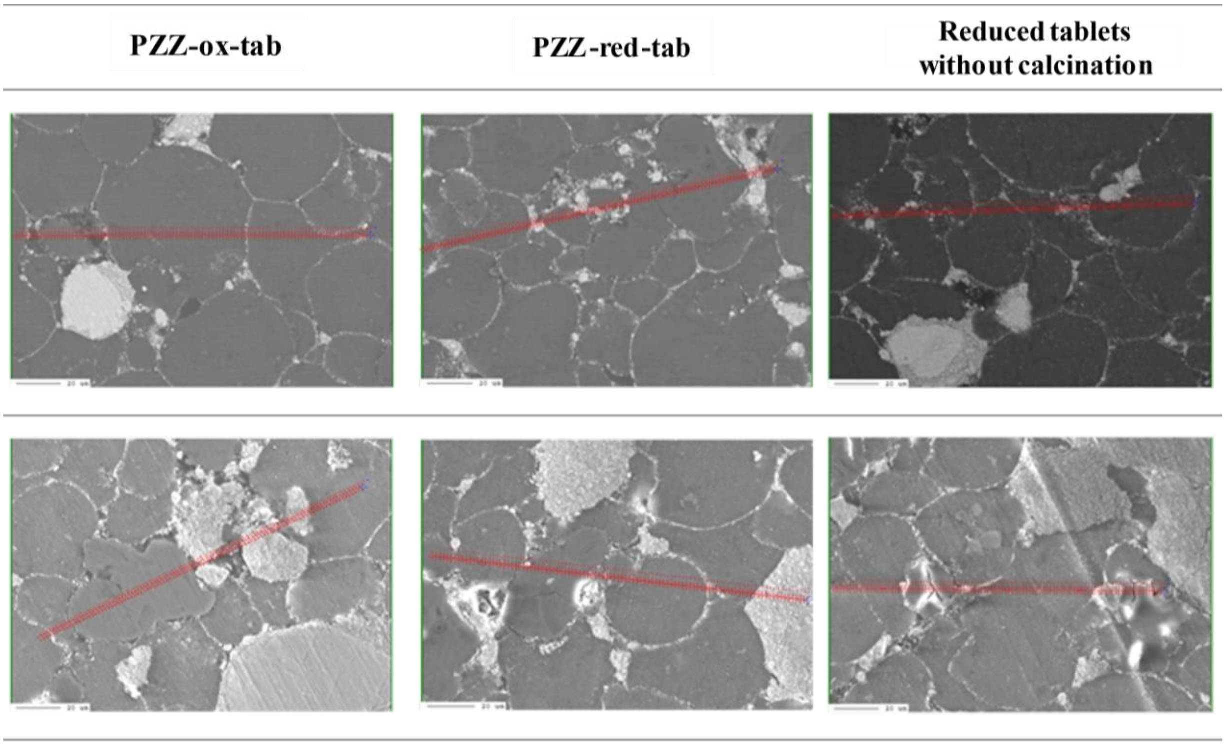

Figure S1. SEM images of PZZ-ox-tab and PZZ-red-tab (calcined and uncalcined). Areas rich in Zr (and Pd) appear as light grey.

Table S2. XANES LCF results for all scaled-up catalysts.

Sample	R-factor	Zn(10%)Al_2O_3 (wt%)	ZnO (wt %)
PZZ-ox-tab	9.4 E-04	12.9 ± 0.9	87.7 ± 0.9
PZZ-red-tab	1.9 E-03	19.6 ± 1.3	80.0 ± 1.3
PZZ-ox-ext	1.4 E-03	77.2 ± 1.2	23.4 ± 1.2
PZZ-red-ext	1.3 E-03	79.3 ± 1.1	20.9 ± 1.1

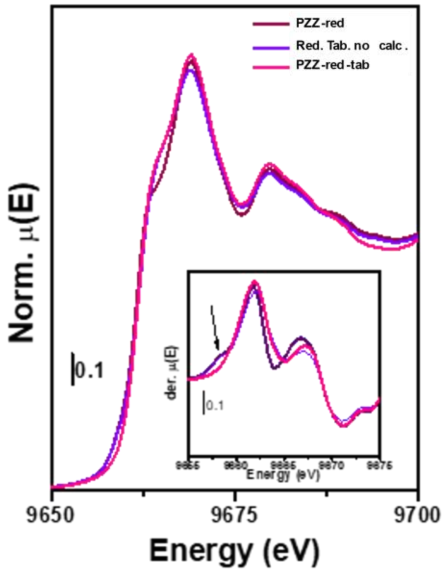

Figure S2. Zn K-edge XAS spectra for PZZ-red (dark red line), reduced tablets without calcination (purple line) and PZZ-red-tab (pink line) catalysts. Spectra first derivative are reported in the inset. PdZn alloy energy shift fingerprint is indicated.

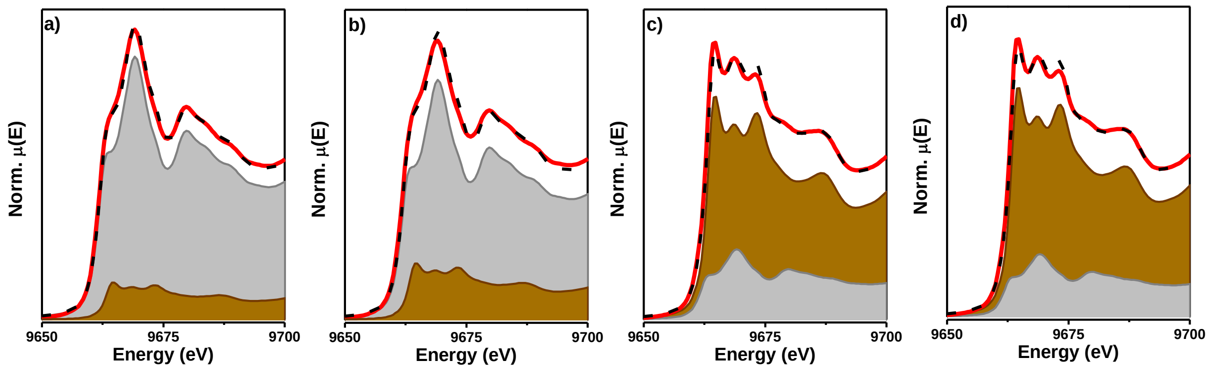

Figure S3. Zn K-edge Linear Combination Fit curve (dashed black line) using ZnO (grey line) and Zn(10%)-Al_2O_3 (brown line) for experimental spectra (red line) of a) PZZ-ox-tab, b) PZZ-red-tab, c) PZZ-ox-ext and d) PZZ-red-ext. Reported ZnO and Zn(10%)Al_2O_3 reference have been weighted to the respective weight percentage resulting from LCF.
